# A Fireworks Algorithm Based on Transfer Spark for Evolutionary Multitasking

**DOI:** 10.3389/fnbot.2019.00109

**Published:** 2020-01-17

**Authors:** Zhiwei Xu, Kai Zhang, Xin Xu, Juanjuan He

**Affiliations:** ^1^School of Computer Science and Technology, Wuhan University of Science and Technology, Wuhan, China; ^2^Hubei Province Key Laboratory of Intelligent Information Processing and Real-Time Industrial System, Wuhan, China

**Keywords:** evolutionary multitasking, multitask optimization, fireworks algorithm, transfer spark, evolutionary algorithm

## Abstract

In recent years, lots of multifactorial optimization evolutionary algorithms have been developed to optimize multiple tasks simultaneously, which improves the overall efficiency using implicit genetic complementarity between different tasks. In this paper, a novel multitask fireworks algorithm is proposed with novel transfer sparks to solve multitask optimization problems. For each task, some transfer sparks would be generated with adaptive length and promising direction vector, which are very helpful to transfer useful genetic information between different tasks. Finally, the proposed algorithm is compared against some chosen state-of-the-art evolutionary multitasking algorithms. The experimental results show that the proposed algorithm provides better performance on several single objectives and multiobjective MTO test suites.

## Introduction

Traditional evolutionary algorithms aim to find the optimal solution for a single optimization problem by applying the reproduction and selection operators to generate better individuals iteratively (Coello et al., 2006). With the complexity of the problem increasing, simultaneously solving multiple optimization problems efficiently and quickly becomes an urgent problem (Ong and Gupta, 2016). In this context, inspired by multitasking learning in the machine learning field (Chandra et al., 2017), evolutionary multitasking (EMT) is proposed to solve the multitask optimization (MTO) problem by encoding the solutions from different tasks into a unified search space and utilizing the information of potential complementarity and similarity of different tasks to improve the convergence speed and the quality of the solutions (Gupta et al., 2016b).

The best known and the first instructive work in the EMT area is the multifactorial evolutionary algorithm (MFEA) (Gupta et al., 2016b, 2017). The MFEA algorithm is inspired by the multifactorial inheritance (Rice et al., 1978; Cloninger et al., 1979). Each task corresponds to a cultural bias block, and each cultural bias block will have an impact on the development of the offspring. When individuals with different cultural biases hybridize, they exchange information about each other's cultures and promote optimization by exploiting the potential genetic complementarity between multiple tasks (Gupta and Ong, 2016). Intuitively, an inferior solution of a task may be an exceptional solution for the other task. Similarly, the same solution in a unified space can also be excellent in multiple tasks concurrently. In both cases, the MFEA allows multiple tasks to bundle together to optimize and share genetic information to improve the overall efficiency of the search process (Gupta et al., 2018). To this end, MFEA also proposed the mechanisms of assortative mating and vertical cultural transmission to ensure the efficiency and intensity of information exchange between tasks. These ideas have a profound impact on subsequent algorithms.

Currently, the research on EMT can approximately be summarized into three categories, the practical application of EMT (Sagarna and Ong, 2016; Yuan et al., 2016; Zhou et al., 2016; Cheng et al., 2017; Binh et al., 2018; Thanh et al., 2018; Lian et al., 2019; Wang et al., 2019) and the improved algorithm based on the MFEA framework (Bali et al., 2017; Feng et al., 2017; Wen and Ting, 2017; Joy et al., 2018; Li et al., 2018; Tuan et al., 2018; Zhong et al., 2018; Binh et al., 2019; Liang et al., 2019; Yin et al., 2019; Yu et al., 2019; Zheng et al., 2019; Zhou et al., 2019) and the perfection of EMT theory (Gupta et al., 2016a; Hashimoto et al., 2018; Liu et al., 2018; Zhou et al., 2018; Bali et al., 2019; Chen et al., 2019; Feng et al., 2019; Huang et al., 2019; Shang et al., 2019; Song et al., 2019; Tang et al., 2019). From the above studies, a consensus can be summarized that efficiently utilizing the inter-task related information is the key to improve overall search efficiency in EMT. Therefore, many studies focus on analyzing and optimizing knowledge transfer between tasks. Zhong et al. (2018) proposed a multitask genetic programming algorithm, which adopted a novel scalable chromosome representation to allow cross-domain coding of multiple solutions in a unified representation. The improved evolutionary mechanism takes both the implicit transfer of useful features between tasks and the ability of exploration into account. Liang et al. (2019) introduced genetic transform strategy and hyper-rectangle search strategy to the MFEA to improve the efficiency of knowledge transfer between tasks in the late iteration of the traditional MFEA. Huang et al. (2019) proposed an efficient surrogate-assisted multitask evolutionary framework with adaptive knowledge transfer, which is very superior for solving expensive optimization tasks. The surrogate model is constructed according to the historical search information of each task and reduces the evaluation times. A universal similarity measurement mechanism and an adaptive knowledge transfer mechanism are proposed to help knowledge transfer efficiently. Chen et al. (2019) presented the adaptive selection mechanism to evaluate the correlation between tasks and cumulative return on knowledge transferring to select the appropriate assisted task for a given task to prevent the influence of negative tasks. Feng et al. (2019) proposed an explicit genetic transferring EMT algorithm by autoencoding. This explicit genetic transfer method effectively utilizes multiple preferences embedded in different evolutionary operators to improve search performance. Bali et al. (2019) adopted the online learning mechanism into EMT and initiated a data-driven parameter tuning multitasking approach to mitigate harmful interactions between unrelated tasks to enhance overall optimization efficiency.

It is noted that most of the existing EMT algorithms are affected by the well-known MFEA algorithm. Individuals exchange genetic information through the chromosomal crossover. The hybridization of individuals with the same cultural background contributes to exploit, while individuals from different cultural backgrounds share information about their respective tasks. However, there are two drawbacks. First, the crossover sites and offset directions are randomly generated; therefore, the information transferred from the other task might not necessarily contribute to the optimization of the target task. Second, the intensity of information exchange is artificially set, and the optimization performance lacks effective feedback on it, which makes the search effect of EMT algorithm sensitive to the relationship between the tasks optimized simultaneously.

Swarm intelligence algorithms have the potential to transfer potential genetic information between tasks due to their inherent parallelism (Feng et al., 2019; Song et al., 2019). Inspired by coevolution (Cheng et al., 2017), by mapping multiple tasks into different subpopulations, the same type of subpopulations compete with each other, and subpopulations with different types cooperate, and potentially helpful knowledge blocks can be efficiently transferred between populations and utilized. The fireworks algorithm (FWA) (Tan and Zhu, 2010) is a recently proposed evolutionary algorithm based on swarm intelligence. First, a fixed number of positions in the search space are chosen as fireworks. Then, a set of sparks is generated through the explosion operation from the fireworks. Afterward, the superior solutions from the whole fireworks and sparks are selected as the fireworks for the next generation to continually improve the quality of the solution iteratively. Benefiting from the powerful global search and information utilization capabilities of FWA, it has attracted much research interest (Zheng et al., 2013; Liu et al., 2015; Li et al., 2017; Li and Tan, 2018) and has demonstrated excellent performance in many real-world problems (Yang and Tan, 2014; Bacanin and Tuba, 2015; Bouarara et al., 2015; Ding et al., 2015; Rahmani et al., 2015). In this paper, an innovative transfer vector (TV) is introduced to represent the bias of knowledge transfer between tasks. The TV is constructed by the current fitness information of other tasks and has promising direction and adaptive length. A potential superiority solution with the probability to navigate other tasks called transfer spark (TS) is generated by adding the TV as the bias to the current firework. A novel multitask optimization fireworks algorithm (MTO-FWA) utilizing the TS to exchange implicit information between tasks is proposed.

The rest of this paper is organized as follows. Section Preliminary introduces the basics of MTO and the benchmark EMT algorithm MFEA. Section Method describes the basic FWA algorithm, the proposed MTO-FWA, and the promotion of MTO-FWA on multiobjective optimization problems. Section Experiments demonstrates the experiment results on both single-objective and multiobjective MTO problems to assess the effectiveness of MTO-FWA. Finally, Section Conclusion concludes this paper and elaborates on future work.

## Preliminary

The section presents the key concept of MTO and the benchmark EMT algorithm MFEA.

### Multitask Optimization

In general, conventional optimization problems can be divided into two categories: single-objective optimization (SOO) problems and multiobjective optimization (MOO) problems (Liang et al., 2019). They are both committed to seeking the optimal solution of an optimization task. The difference is that SOO has only one objective function, while MOO needs to optimize multiple conflicting objective functions. The purpose of the SOO is to search out the solution with the best function value, while the goal of the MOO problem is to obtain a solution set with splendid convergence and diversity. Inspired by the cognitive ability of humans to multitasking, the knowledge acquired from solving the problem can enlighten the optimization of related problems (Gupta et al., 2016b). MTO is devoted to implementing an evolutionary search on multiple optimization tasks simultaneously to improve the convergence by seamlessly transferring knowledge between multiple optimization problems.

**Figure 1:**
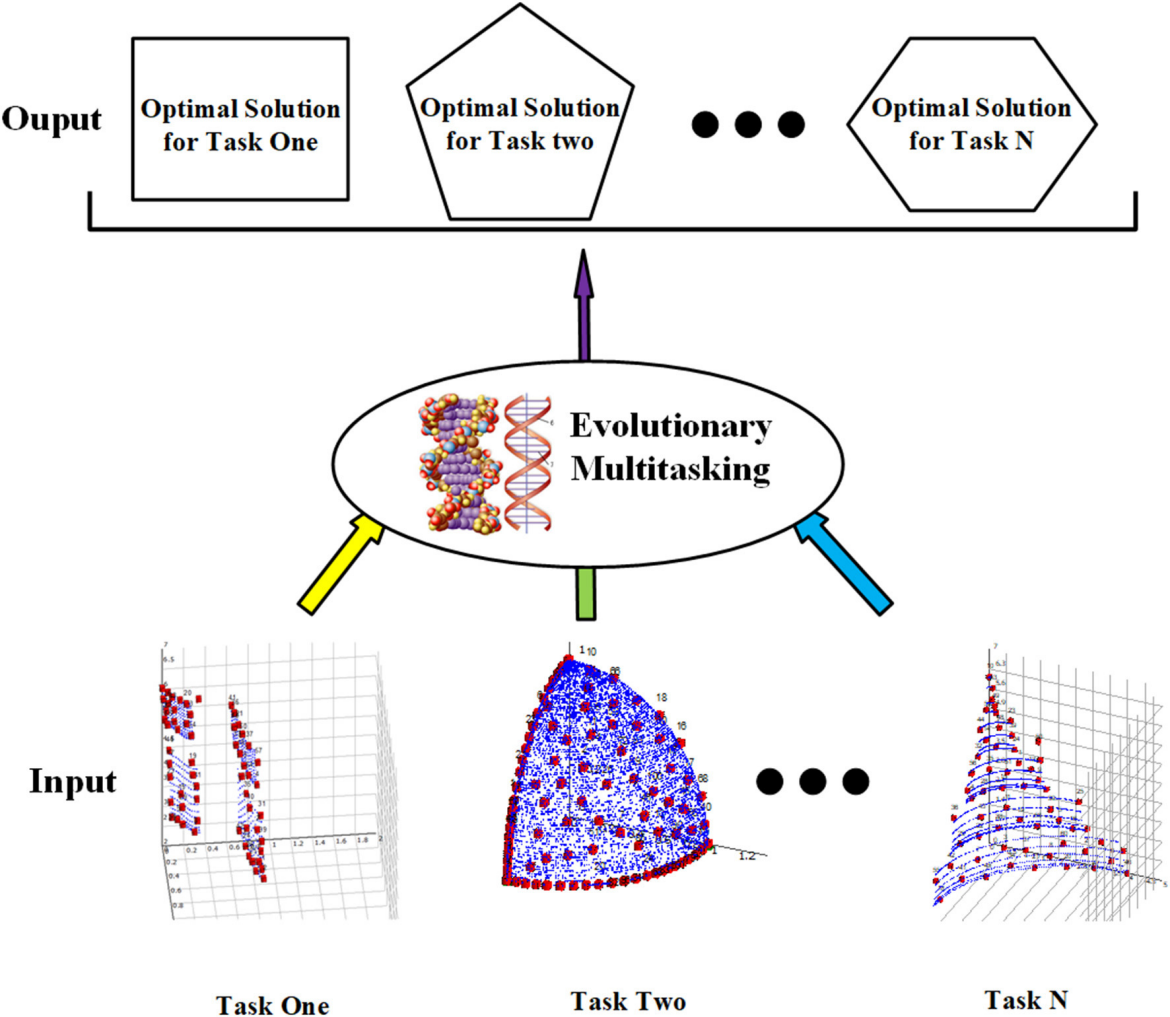
Illustration of multitask optimization.

Unlike SOO and MOO, MTO is a new paradigm that aims to seek out the optimal solutions for multiple tasks at once. As shown in Figure 1, the input to the MTO consists of multiple optimization tasks, each of which can be a SOO or MOO problem. All the tasks are handled by the MTO paradigms simultaneously, so the output of the MTO contains the optimal solution for each task separately.

(1) $$\begin{array}{l} \left\{X_{1},X_{2},\cdots \;,X_{K}\right\}\\ \;=\left\{\text{argmin}T_{1}\left(X_{1}\right),\text{argmin}T_{2}\left(X_{2}\right),\cdots \;,\text{argmin}T_{K}\left(X_{K}\right)\right\} \end{array}$$

The formal representation of MTO is shown in formula (1), where *X*_*j*_ denotes the optimal solution of the *j*th task *T*_*j*_ (*j* = 1,2,…*K*).

### Multifactorial Evolutionary Algorithm

Inspired by the multifactorial inheritance (Rice et al., 1978; Cloninger et al., 1979), a novel EMT paradigm multifactorial optimization is proposed. Each task *T*_*j*_ is considered as a factor affecting individual evolution in the *K*-factorial environment [4, 5]. MFEA is a popular implementation that integrates genetic operators in genetic algorithm into multifactorial optimization (Gupta et al., 2016b, 2017; Bali et al., 2017; Feng et al., 2017; Wen and Ting, 2017; Binh et al., 2018, 2019; Li et al., 2018; Thanh et al., 2018; Zhong et al., 2018; Zhou et al., 2018, 2019; Liang et al., 2019; Shang et al., 2019; Yin et al., 2019; Yu et al., 2019; Zheng et al., 2019). All the individuals are encoded into a unified search space *Y*, and each individual can be decoded to optimize different component problems to effectively realize cross-domain knowledge transfer. In general, *Y* is normalized to [0, 1]^*D*^, where *D* is the number of dimensions of the unified search space. *D* = max {*D*_*j ϵ* {1, 2, …*K*}_}, where *D*_*j*_ indicates the number of dimensions of the *j*th task. By coding, a single chromosome *y* ∈ *Y* can signify a combination of chromosomes corresponding to *K* different tasks. By decoding, the chromosomes in the unified search space can be differentiated into *K* chromosomes specific to the task. To evaluate the performance of a solution in the uniform search space on different tasks, MFEA proposes some definitions.

Factorial Cost: The factorial cost of individual *p*_*i*_ is defined as $\psi_{j}^{i}$ which is applied to measure the performance of individual *p*_*i*_ on a specific task *T*_*j*_. When the *p*_*i*_ is the feasible solution of task *T*_*j*_ and satisfies the constraint conditions, $\psi_{j}^{i}$ is the fitness value of *T*_*j*_. Otherwise, $\psi_{j}^{i}$ is a very large value and indicates that the individual *p*_*i*_ is not a candidate solution of task *T*_*j*_.

Factorial Rank: The factorial rank $r_{j}^{i}$ indicates the rank of fitness values $\psi_{j}^{i}$ for an individual *p*_*i*_ on a given task *T*_*j*_ by sorting the $\psi_{j}^{i}$ in ascending order.

Scalar Fitness: To illustrate the best performance that an individual can achieve in all tasks. The scalar fitness φ_*i*_ is defined based on the best factorial rank of individual *p*_*i*_ among all the tasks that can be expressed as φ_*i*_ = $\frac{1}{min_{j\;\epsilon \;\left\{1,2\ldots k\right\}}r_{j}^{i}}$.

Skill Factor: The skill factor τ_*i*_ of individual *p*_*i*_ represents the task that *p*_*i*_ shows the best performance, which is defined as $\tau_{i}=argmin_{j}\left\{r_{j}^{i}\right\}$.

Besides the traditional genetic operators, MFEA also applies the assortative mating to control the strength of genetic information transfer between tasks and vertical cultural transmission to enhance the efficiency of implicit knowledge transfer.

Assortative Mating: For two randomly selected individuals, if their skill factor is the same or satisfied the threshold called random mating probability (RMP), they can perform crossover to exchange their respective genetic information or they can only mutate. Intuitively, individuals with the uniform skill factor have a high probability of performing the crossover operator but individuals from different tasks can only exchange their genetic information in a small probability.

Vertical Cultural Transmission: Inspired by the multifactorial inheritance, MFEA believes that offspring will share the same cultural environment with their parents; that is, offspring should inherit their skill factors from their parents. If the offspring is obtained by the crossover operator, it will inherit the skill factor of either parent with equal probability. Otherwise, if the offspring is generated by the mutation operator, its skill factor will be completely inherited from the only parent. Based on the previous definitions, the pseudocode of the basic MFEA algorithm is shown in Algorithm 1.

**Algorithm 1:** The Pseudocode of MFEA

*N*, the size of population;
*K*, the number of the optimization tasks;
Randomly generate *N* individuals as the initial population *P*.
Assign initial skill factor to each individual in *P* randomly.
Evaluate the factorial cost of each individual
while the maximum number of evaluations is not reached:
Generate the offspring population *Q* according to assortative mating mechanism.
Offspring inherit the skill factor based on vertical cultural transmission strategy.
Evaluate individuals in *Q*.
Merge *P* and *Q* to generate new population *R* = *P*⋃*Q*.
Update the scalar fitness φ and skill factor τ of every individual in *R*.
Select the fittest *N* individuals from *R* as the new *P*.
end while

## Methods

This section introduces the basic FWA, the MTO-FWA based on the TS, and the extended multiobjective MTO-FWA.

### The Basic FWA

Illuminated by the phenomenon that fireworks exploding to generate some explosion sparks and illuminate a surrounding area, a novel swarm intelligence algorithm FWA is proposed (Tan and Zhu, 2010). It believes that the fireworks explosion phenomenon is analogical to the process of searching the optimal solution. If there is a promising area around the current search space, fireworks will migrate to that area and generate explosion sparks to perform the local search.

The prime procedure of FWA is as follows: first, randomly initialize a set of fireworks and evaluate each firework according to the objective function Then, each firework performs a local search through an explosion operation. To save computational resources and improve search efficiency, the resource allocation strategy is used to allocate the scope and frequency of each fireworks local search. In general, individuals with better fitness function values are considered more likely to lead to global optimum, and therefore are allocated more search resources. Based on the above ideas, fireworks with better fitness values will generate a mass of sparks and possess smaller explosion amplitudes, and fireworks with worse fitness values can only generate a smaller amount of sparks and have wider explosion amplitudes relatively. After the explosion, the Gaussian mutation operation is applied to produce Gaussian mutation sparks to increase the diversity of the population. Finally, the next generation of fireworks is selected from the candidate set including fireworks, and the sparks produced by explosion and Gaussian mutation based on their performance. The processes repeat iteratively until the maximum number of evaluations is reached.

#### Explosion Operation

In the basic FWA algorithm (Tan and Zhu, 2010), the number of sparks and explosion amplitude of each firework *x*_*i*_ are shown in formula (2) and (3), respectively:

(2) $$\begin{array}{l} S_{i}=\hat{S}\cdot \frac{f_{max}-f\left(x_{i}\right)+\epsilon }{\sum_{i\;=\;1}^{N}\left(f_{max}-f\left(x_{i}\right)\right)+\epsilon } \end{array}$$

(3) $$\begin{array}{l} A_{i}=\hat{A}\cdot \frac{f\left(x_{i}\right)-f_{min}+\epsilon }{\sum_{i\;=\;1}^{N}\left(f\left(x_{i}\right)-f_{min}\right)+\epsilon } \end{array}$$

where $\hat{S}$ and $\hat{A}$ are two artificial parameters to control the total number of fireworks and the total amount of explosion amplitude, respectively, *N* represents the population size, *f*_max_ and *f*_min_ denote the maximum and minimum objective values among the total fireworks, and ϵ indicate a tiny real value to prevent zero as the denominator. To avoid this, good fireworks have too many explosion sparks, but bad fireworks have very few explosion sparks. Two other constants parameters *a, b* ∈ [0,1] are introduced to bound the *S*_*i*_ to a proper range.

(4) $$\begin{array}{l} S_{i}=\{\begin{array}{l} round\;\left(a\cdot \hat{S}\right),\;x<a\cdot \hat{S}\\ round\;\left(b\cdot \hat{S}\right),\;x>b\cdot \hat{S}\\ round\;\left(\hat{S}\right),\;otherwise \end{array} \end{array}$$

Conventional FWA does not conduct the explosion operation on each dimension of fireworks, but randomly selects *D*_*explosion*_ dimensions for explosion operation. Each dimension *d* of explosion spark *e*_*is*_, which can be indicated as $e_{is}^{d}$ with *s* ∈ [1, *S*_*i*_], *d* ∈ [1, *D*_*explosion*_], conducts explosion operation according to formula (5).

(5) $$\begin{array}{l} e_{is}^{d}=x_{i}^{d}+A_{i}\cdot random\left(-1,1\right) \end{array}$$

The spark generated by the explosion may exceed the boundary of the search space. FWA proposed the mapping rule to map it back to the search space as expressed in formula (6).

(6) $$\begin{array}{l} e_{is}^{d}=x_{min}^{d}+e_{is}^{d}mod\left(x_{max}^{d}-x_{min}^{d}\right) \end{array}$$

The outline of the explosion process is provided in Algorithm 2.

**Algorithm 2:** The Pseudocode of explosion

for *s* = 1 → *S*_*i*_ do
Initialize the explosion spark: *e*_*is*_ = *x*_*i*_
*D*_*explosion*_ = *round*(*D*·*random*(0, 1))
Stochastically choose *D*_*explosion*_ dimensions of *e*_*is*_.
for each dimension *d* of *D*_*explosion*_ dimensions do
$e_{i}s^{d}=e_{i}s^{d}+A_{i}\cdot random\left(-1,1\right)$
if $e_{is}^{d}$ is out of the threshold value then
$e_{is}^{d}=x_{min}^{d}+e_{is}^{d}mod\left(x_{max}^{d}-x_{min}^{d}\right)$
end if
end for
end for

#### Gaussian Mutation Operator

Some specific sparks are generated by the Gaussian explosion, which adds an offset that satisfies a Gaussian distribution to the spark to increase the diversity of population. The process of the Gaussian explosion is shown in formula (7).

(7) $$\begin{array}{l} {\overset{⌣}{e}}_{i}^{d}=x_{i}^{d}\cdot Gaussian\left(1,1\right) \end{array}$$

Similar to the explosion process, the Gaussian mutation also randomly selects *D*_*gaussian*_ dimensions to mutate. ${\overset{⌣}{e}}_{i}^{d}$ indicates the *d* dimension of the Gaussian mutation spark with *d* ∈ [1, *D*_*gaussian*_].

#### Selection Mechanism

At each iteration of the algorithm, *N* individuals should be retained for the next generation. The individual with the best fitness is preferentially kept among all the current sparks and fireworks. Then, the remaining *N* – 1 individuals are chosen with the probability that is proportional to their distance from other individuals to maintain the diversity of sparks. Manhattan distance (Chiu et al., 2016) is usually used to measure the distance between a solution with other solutions. The choosing probability of the individual *x*_*i*_ represents as *Pb*(*x*_*i*_) defined in formula (8), where *M* denotes the solution set containing all the current individuals of both fireworks and sparks.

(8) $$\begin{array}{l} Pb\left(x_{i}\right)=\frac{Manhattan\;distance\left(x_{i}\right)}{\sum_{i\;\in \;M}Manhattan\;distance\left(x_{i}\right)} \end{array}$$

#### The Structure of the FWA

Algorithm 3 summarizes the FWA framework. After the fireworks explode, the explosion sparks and Gaussian mutation sparks are generated based on Algorithm 2 and formula (7), respectively. The explosion sparks are generated according to the explosion operator, and the number and amplitude of the spark depend on the fitness of the firework. The Gaussian mutation sparks are generated by the Gaussian explosion process, whose number is denoted by *Gas*. Finally, *N* individuals remain for the next generation according to the selection mechanism.

**Algorithm 3:** The Pseudocode of FWA

*N*, the size of population;
*Gas*, the number of Gaussian mutation spark;
Randomly generate *N* initial fireworks.
while the maximum number of evaluations is not reached:
for each firework *x*_*i*_ do
Calculate the number of sparks *S*_*i*_ and the explosion amplitude *A*_*i*_ according to formula (2) and (3).
Generate explosion sparks of the firework *x*_*i*_ based on Algorithm 2.
end for
for *gas* = 1:*Gas* do
Obtain a Gaussian mutation spark for a randomly selected firework *x*_*j*_ using formula (7).
end for
Evaluate all the fireworks and sparks.
Select *N* suitable solutions to constitute the fireworks of next iteration according to the selection mechanism.
end while

### Multitask Optimization Firework Algorithm

For MTO problems, the objective function landscape is heterogeneous, and the worst case is that they are not similar or intersecting. The key of EMT is to effectively utilize the implicit genetic information complementation from different tasks to improve the overall efficiency. Therefore, the interaction and transfer of information between different tasks are very important.

Swarm intelligence algorithms frequently possess multiple populations, which can grow the cognition of search space and further the diversity of solutions. This is very promising for exploring the heterogeneous search space of MTO problems. Different tasks can be assigned to different populations, and the cooperation between different populations provides an interpretable theoretical basis for information interaction between tasks. Different from the crossover process of randomly selected individuals in MFEA, information interaction between populations utilizes information from the whole population, which can effectively avoid random noise and negative knowledge transfer.

Unlike other swarm intelligence algorithms, FWA naturally possesses multiple populations on account of that every spark is generated near its parent firework and therefore they have similar properties. Just based on such an evolutionary strategy, each firework and its generated sparks are constituted as a task module, and each one is allocated a specific task. Disparate task modules exchange information to facilitate the exchange of implicit genetic information and individuals within a module compete with each other to promote convergence.

Compared with the conventional FWA, the main motivation of MTO-FWA can be summarized as two points.

**Figure 2:**
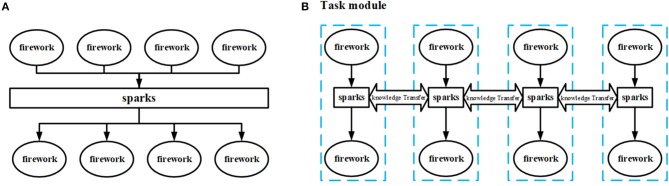
**(A)** The framework of conventional FWA vs. **(B)** the framework of MTO-FWA.

1. Combine fireworks and their sparks into a task module to solve a specific task. Competition comes from within modules, and communication between tasks is based not on individuals but the module population. The comparison between the task module structure and the conventional FWA structure is shown in Figure 2.
2. A TS is proposed to solve information transfer and knowledge reuse between different tasks.

#### Explosion Operation

The traditional method controlling the number of sparks is sensitive to the maximum fitness value in the population, and the resource allocation gap between individuals is uncontrollable. The individuals with the highest adaptive value may get all the resources, while those with the lowest adaptive value may not get any resources. The traditional FWA solves this problem by setting thresholds, but this is crude and inelegant. Therefore, we use the power-law distribution (Li et al., 2017) to allocate spark number, through fitness rank rather than the fitness value to determine the number of spark explosion fireworks, which is shown in formula (9).

(9) $$\begin{array}{l} S_{r}=\hat{S}\cdot \frac{r^{-\alpha }}{\sum_{r\;=\;1}^{N}r^{-\alpha }} \end{array}$$

*N* represents the total number of fireworks, *r* denotes the fitness rank of fireworks, and α indicates the artificial parameter controlling the distribution of resource allocation. The larger the α, more explosion sparks a good firework produces.

For the amplitude, the dynamic control algorithm (Li et al., 2017) is used, and the explosion amplitude of all fireworks is controlled dynamically, as shown in formula (10).

(10) $$\begin{array}{l} f\left(x\right)=\{\begin{array}{ll} A_{i}^{1}, & \;g=1\\ C_{r}A_{i}^{g-1}, & \;f(x_{i}^{g})⩾f(x_{i}^{g-1})\\ C_{a}A_{i}^{g-1}, & \;f\left(x_{i}^{g}\right)<f(x_{i}^{g-1}) \end{array} \end{array}$$

where $A_{i}^{g}$ denotes the explosion amplitude of the *i*th firework in generation *g*. In the initialization generation, the explosion amplitude is set to a large real value, usually the diameter of the search space. If the function value of the offspring firework is larger than that of the parent fireworks, the explosion amplitude will be multiplied by a shrink coefficient *C*_*r*_ < 1 to reduce the explosion amplitude so as to exploit a better solution in the local scope. Instead, the amplitude of the explosion is multiplied by an amplification coefficient *C*_*a*_ > 1 to attempt to make the largest progress. In other words, the explosion amplitude is very large at the beginning of the iteration and shrinks to a smaller value in the later stages of the iteration by the dynamic tuning strategy.

It should be emphasized that the proposed MTO-FWA has the same mapping rules as FWA. The difference is that the explosion operator works in each dimension of fireworks instead of the *D*_*explosion*_ dimensions randomly selected, which has been proven to be more effective than the method of randomly selected dimensions (Li and Tan, 2018).

#### Guiding Spark

Different from the conventional FWA, the proposed MTO-FWA uses the guiding spark (GS) (Li et al., 2017) instead of the Gaussian mutation operator. The GS can guide the fireworks in a good direction by adding a guiding vector that indicates the dominant direction and step size to the fireworks location. The guiding vector is obtained by calculating the average of the differences between the pre-σ*S*_*i*_ sparks and the post-σ*S*_*i*_ sparks after all the sparks are sorted by their fitness values *f* (*e*_*is*_) in the ascending order. By using the deviation between the top population and the bottom population, the random noise can be effectively reduced, the fireworks can be guided in the right direction, and the step length can be adjusted adaptively with the distance from the minimum value of the objective function. The generation of GS for the *i*th firework is shown in formula (11).

(11) $$\begin{array}{l} \Delta_{i}=\frac{1}{\sigma S_{i}}\left(\sum_{s\;=\;1}^{\sigma S_{i}}e_{is}-\sum_{s\;=\;S_{i}-\sigma S_{i}+\;1}^{S_{i}}e_{is}\right)\\ GS_{i}=x_{i}+\Delta_{i} \end{array}$$

where σ is the ratio parameter, *e*_*is*_ represents the *s*th explosion spark generated by the *i*th fireworks, Δ_*i*_ indicates the guiding vector of the *i* th fireworks, and *GS*_*i*_ denotes the GS of the *i* th fireworks. It is worth noting that only one GS is generated for each firework.

#### Transfer Spark

The TS is proposed to exchange information between different tasks in MTO-FWA. Each firework, explosion spark, and GS will be assigned a skill factor, and the spark inherits the skill factor from their parents. The firework and its sparks constitute a task module with the same skill factor. To avoid excessive evaluations, individuals will only evaluate the fitness values of the tasks they are assigned. In the MTO problem, according to the concept of implicit genetic information complementation, the location information of a task module can greatly help optimize another task. Based on this, assume the *i*th firework for the optimization task *j* denoted as $FW_{j}^{i}$, it generates a unique spark for optimizing the task *k* according to the information from the task *k*. This information from task *k* is denoted as $TV_{jk}^{i}$. This spark is different from other sparks generated by $FW_{j}^{i}$ as its skill factor is *k*. Since it can transfer the information from other tasks, this type of spark is named TS. The TS generated by $FW_{j}^{i}$ under the guiding of $TV_{jk}^{i}$ is represented as $TS_{jk}^{i}$. $TV_{jk}^{i}$ and $TS_{jk}^{i}$ can be obtained by equations (12) and (13), respectively.

(12) $$\begin{array}{l} TV_{jk}^{i}=\frac{2}{\sigma M_{j}+\sigma M_{k}}\left(\sum_{i\;=\;1}^{\sigma M_{k}}x_{k}^{i}-\sum_{i\;=\;1}^{\sigma M_{j}}x_{j}^{i}\right)\;\frac{r^{-\alpha }}{\sum_{r\;=\;1}^{N_{j}}r^{-\alpha }} \end{array}$$

(13) $$\begin{array}{l} TS_{jk}^{i}=FW_{j}^{i}+TV_{jk}^{i} \end{array}$$

where *M*_*k*_ and *M*_*j*_ denote the total number of the individuals that the skill factor is *k* and *j*, respectively. In general, *M*_*k*_ is equal to *M*_*j*_. σ*M*_*k*_ represents the best σ*M*_*k*_ th individuals in ascending order of fitness value of task *k*, and σ*M*_*j*_ indicates the best σ*M*_*j*_ th individuals of task *j*. The average value of the difference of each of the best σ*M*th individuals is taken as a deviation. Then, each firework will be assigned deviation using the power-law distribution according to the fitness rank. The fireworks that perform better on task *j* are considered to have more genetic advantages and will be given more information from task *k*. In contrast, individuals who perform poorly on the original task can only be assigned a small amount of exchanged genetic information.

Conventional EMT algorithms randomly select individuals with different skill factors to crossover for genetic information transfer. In FWA, the locations and fitness values of the sparks generated by the explosion contain a lot of information about the objective function. Even the inferior solution that will be eliminated in the selection process still contains the genetic information that can play a great positive role in understanding the fitness landscape of the objective function and transferring the genetic information between tasks. In general, this information is ignored and not effectively utilized. Given this, we use dominant subpopulations rather than a single optimal individual for transferring genetic information in MTO. Second, by using subpopulations for information transfer, the uncorrelated values will be canceled out. Most of the dimensions of the best spark are good, but the rest are not, which means that to learn from the single best individual is to learn its good and bad at the same time. However, learning from a good population is another matter. Only the common characteristics of the population will be transferred, and other information will be regarded as random noise canceling each other, so the transferred knowledge will be more accurate.

Most EMT algorithms use crossover operators to transfer knowledge between tasks, such as SBX crossover operators. The idea is to do a local search around the parents from different tasks, and most of the offspring will fall closer to their parents, and a few will fall in between. TV, which is essentially a similar effect, can be thought of as the average of the σ*M* vectors pointing from task *j* to task *k*, and the generated solution *TS*_*jk*_ will fluctuate between the superior subpopulations of *x*_*j*_ and *x*_*k*_.

#### Selection Mechanism

All the individuals in the same task module have the same skill factor, and an individual with the best fitness in a task module is kept as candidate firework, instead of selecting from the entire individual pool. Then, all candidate fireworks and TSs are then combined and grouped according to skill factor. Afterward, the selection probability is assigned according to the fitness value of the individual, and each group will select *N* solutions according to this probability as the next generation of fireworks. For task *j*, the selection strategy is shown in Algorithm 4.

**Algorithm 4:** Selection mechanism

*N*_*j*_, the population size of task *j*;
Keep the individual with the best fitness in each task module with skill factor τ_*j*_ as the candidate solution.
Merge the candidate solutions and the all the TS with skill factor τ_*j*_ as set *U*_*j*_.
Assign the selection probability of the solution in *U*_*j*_ according to factorial rank $r_{j}^{i}$.
Select *N*_*j*_ solutions in *U*_*j*_ according to the selection probability as the fireworks in the next generation.

#### The Structure of the MTO-FWA

Algorithm 5 summarizes the MTO-FWA framework. Assume that *K* tasks are optimized simultaneously; first, all the fireworks are initialized randomly and each one is evaluated by all the tasks. Then, each firework is assigned a skill factor τ according to their performance. After a firework exploding, *S*_*i*_ sparks with different explosion amplitude *A*_*i*_ are generated according to formulas (9) and (10). After that, a GS is generated by using the knowledge of exploding fireworks according to formula (11), and the skill factors of the explosion sparks and the GS are all set to τ. Afterward, *K*−*1* TS are generated for other *K*−*1* tasks, respectively, to share knowledge according to formulas (12) and (13). Finally, each task applies the selection strategy to pick the appropriate solutions for the next generation according to Algorithm 4.

**Algorithm 5:** The overall framework of MTO-FWA

Randomly initialize fireworks.
Evaluate the objective values of different tasks for each firework.
Assign skill factor τ to each firework according to the fitness value
while not reach stop criteria
for each firework *x*_*i*_ do
Calculate the number of sparks *s*_*i*_ according to formula (9), the explosion
amplitude *A*_*i*_ based on formula (10).
Obtain locations of explosion sparks of the firework
*x*_*i*_ and assign skill factor τ.
Generate a GS according to formula (11) and assign skill factor τ.
for each remaining *K-1* tasks do
Produce a TS according to formula (12) and (13),
then assign skill factor $\overset{⌣}{\tau }$.
end for
end for
for each task do
Select the solutions for the next generation according to Algorithm 4.
end for
end while

### Multitask Optimization Firework Algorithm for MOO

Multiobjective problems have two or more conflicting objectives for simultaneous optimization. Due to the lack of prior knowledge of the objective functions, we always study plentiful obtained solutions and retain the non-dominated solutions, the Pareto solution set, as the approximation of the true Pareto optimal set. Based on the fact that FWA is adept in using a single indicator to conclude the number of explosion sparks and the explosion amplitude, considering that MOO requires both convergence and diversity, the S-metric indicator (Liu et al., 2015) is introduced into FWA instead of the fitness value to select and evaluate the solutions. It should be noted that in the proposed multitask firework algorithm for MOO (MOMTO-FWA), except for the indicator modified to S-metric, the explosion operator, the GS, the TS, and the MTO-FWA are consistent. The following sections highlight the S-metric and the external archive mechanism for preserving non-dominated solutions.

#### S-Metric

The S-metric indicator can be regarded as the size of the space dominated by the solution or solution set (While et al., 2006). The S-metric for a solution set *M* = {*m*_1_, *m*_2_, ⋯*m*_*i*_⋯*m*_*n*_} is indicated as formula (14) (Emmerich et al., 2005).

(14) $$\begin{array}{l} S\left(M\right):=\wedge \left(\underset{m\in M}{⋃}\left\{x|m≺x≺x_{ref}\right\}\right) \end{array}$$

where ∧ denotes the Lebesgue measure, ≺ denotes the dominance relationship, and *x*_*ref*_ indicates the reference point dominated by all the solutions. Homoplastically, the S-metric for a single solution is represented as formula (15).

(15) $$\begin{array}{l} S\left(m_{i}\right)=\Delta S\left(M,m_{i}\right):=S\left(M\right)-S\left(M\backslash \left\{m_{i}\right\}\right) \end{array}$$

The S-metric of a solution *m*_*i*_ can be considered as the region that is only dominated by *m*_*i*_ but not by other solutions in the population.

#### External Archive Mechanism

To ensure the quality of the solution, MOMTO-FWA uses an external archive mechanism to save the advantageous solutions for the entire iteration of each task. The number of individuals in the external archive remains at a fixed value *E*. For a single task *k*, the *E*_*k*_ solutions are selected from a pool of candidates *M*_*k*_ consisting of all fireworks, explosion sparks, GS, and TS with the same skill factor of τ_*k*_. By selecting the optimal solution with the largest S-metric and updating the S-metric of remaining solutions, the selected *E*_*k*_ solutions gain the maximum S-metric in all the *E*_*k*_ sets. It has been proven that the solution set that has the theoretic maximum of S-metric comes necessarily from the True Pareto Front (Fleischer, 2003). The concrete mechanism of update the external archive of the specific task is shown in Algorithm 6.

**Algorithm 6:**
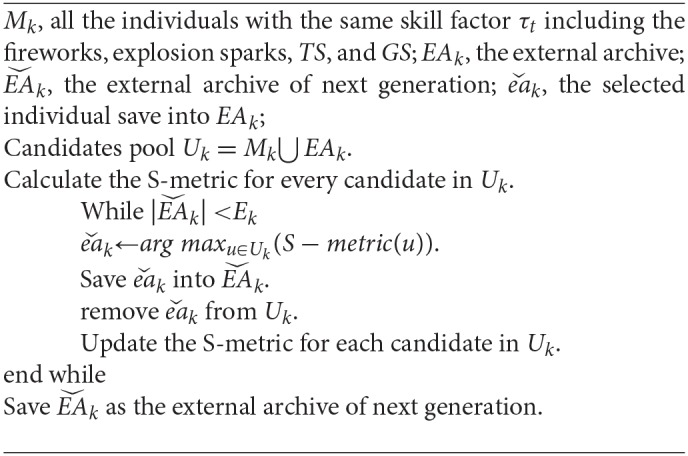
Updating strategy for the external archive

## Experiments

In this section, the proposed MTO-FWA is compared with other state-of-the-art EMT algorithms. The performance of MTO-FWA is comprehensively evaluated by the single-objective MTO test suite and the performance of MOMTO-FWA is assessed by the multiobjective MTO test suite.

### Experiments on MTO for Single-Objective Problems

The performances of EMT algorithms are evaluated by the classical single-objective MTO test suite presented in the evolutionary MTO technical report (Da et al., 2017). The similarity of the fitness landscape and the degree of intersection of the global optima are the two key factors affecting genetic complementarity between different tasks. In other words, if the values of the corresponding dimensions of the global optima of different tasks are closer, the genetic information of the task is more likely to generate complementarity. Homoplastically, the more similar the fitness landscape of the optimization functions of the different tasks, the more helpful the knowledge an individual learns from one task to optimize other tasks indirectly. Therefore, based on the degree of intersection of the global optima, the designed benchmark problems can be divided into complete intersection (CI), partial intersection (PI), and no intersection (NI) categories. According to the similarity in the fitness landscape, the designed benchmark problems can be categorized as High Similarity (HS), Medium Similarity (MS), and Low Similarity (LS) classes. Based on the combination of the above two classification strategies, nine continuous MTO benchmark problems for SOO are proposed, each problem consisting of two classical SOO functions including the Sphere, Rosenbrock, Ackley, Rastrgin, Griewank, Weierstrass, and Schwefel functions.

As a typical swarm intelligence algorithm, the proposed MTO-FWA is compared not only with the classical basic MFEA algorithm but also with MFDE and MFPSO (Feng et al., 2017), the two swarm intelligence EMT algorithms. For a fair comparison, the population number for a single task is set to 100, and the maximum number of fitness evaluation for a single task is set to 100,000, using the average results of 30 independent runs for comparison. The MFEA uses simulated binary crossover operator (SBX) and polynomial mutation methods produce offspring to reproduce offspring, the RMP is set to 0.3, *p*_*c*_ and η_*c*_ in SBX are set to 1 and 2, respectively, and the parameters in polynomial mutation *p*_*m*_ and η_*m*_ are set to 1 and 5, respectively. In MFPSO, the *w* decreases linearly from 0.9 to 0.4; *c1, c2*, and *c3* are all set to 0.2; and the RMP is also set to 0.3. In MFDE, the RMP is set to 0.3, and *F* and *CR* are set to 0.5 and 0.9. To ensure fairness, in MTO-FWA, the RMP is also set as 0.3; *C*_*r*_, *C*_*a*_, σ, and α are set to 0.9, 1.2, 0.2, and 0.

Table 1 shows the average and standard deviation of the objective function values of all algorithms that run 30 times independently on the classical single-objective MTO test suite. The superior average objective value results are highlighted in bold. The Wilcoxon rank sum test is performed at the significance level of 5%, and the proposed MTO-FWA is compared with other EMT algorithms. Significantly better and worse results than the basic MFEA are presented as “+” and “−”.

**Table 1:** Averaged objective value and standard deviation obtained by MTO-FWA, MFPSO, MFDE, and MFEA on the single-objective multitask problem.

		**MTO-FWA**	**MFPSO**	**MFDE**	**MFEA**
CIHS	T1	**4.638E**–**7+** (3.264E−7)	2.147E−1+ (4.836E−2)	9.696E−4+ (3.625E−3)	3.684E−1 (6.462E−2)
	T2	**8.672E**−**5**+ (6.218E−5)	7.865E0+ (3.692E1)	2.256E0+ (7.854E0)	1.875E2 (3.854E1)
CIMS	T1	**8.239E**−**5**+ (1.173E−4)	5.871E−2+ (3.106E−2)	9.872E−4+ (2.765E−3)	4.426E0 (5.832E−1)
	T2	**9.634E**−**6**+ (2.928E−5)	5.938E0+ (2.812E1)	3.672E−3+ (1.361E−2)	2.234E2 (5.364E1)
CILS	T1	**2.316E0**+ (4.176E−2)	5.326E0+ (9.162E0)	2.203E1– (3.851E−2)	2.017E1 (6.797E2)
	T2	1.173E4– (1.161E3)	**2.172E3**+ (4.163E3)	1.183E4– (1.506E3)	3.694E3 (5.361E2)
PIHS	T1	**7.124E1**+ (1.763E1)	2.012E2+ (1.368E2)	7.629E1+ (1.128E1)	5.768E2 (9.744E1)
	T2	**5.647E**−**6**+ (4.293E−6)	3.625E3– 1.367E2	2.196E−5+ (2.861E−5)	9.736E0 (1.852E0)
PIMS	T1	**7.072E**−**4**+ (8.106E−4)	2.953E0+ (3.157E−1)	9.529E−4**+** (8.694E−4)	3.573E0 (5.821E−1)
	T2	8.168E1+ (1.632E1)	1.176E2+ (1.583E2)	**6.654E1**+ (2.216E1)	6.914E2 (3.128E2)
PILS	T1	1.263E−1+ (2.684E−1)	**9.521E−3**+ (5.130E−2)	3.613E−1+ (5.148E−1)	2.001E1 (9.424E−2)
	T2	**3.564E**−**2**+ (6.845E−2)	4.672E−2+ (1.396E−1)	2.175E−1+ (4.673E−1)	1.962E1 (2.765E0)
NIHS	T1	8.521E1+ (3.262E1)	**4.216E1**+ (2.723E1)	8.812E1+ (4.171E1)	9.894E2 (4.328E2)
	T2	2.716E1+ (9.864E0)	3.672E1+ (1.128E2)	**1.976E1**+ (1.493E1)	2.627E2 (7.632E1)
NIMS	T1	**1.184E−3**+ (2.651E−3)	4.691E−1– 2.966E−1	1.987E−3+ (4.282E−3)	4.248E−1 (6.384E−2)
	T2	**2.658E0**+ (1.113E0)	1.332E1+ (1.942E0)	2.968E0+ (1.062E0)	2.772E1 (2.961E0)
NILS	T1	1.012E2+ (2.106E1)	3.167E2+ (1.176E2)	**9.478E1**+ (1.971E1)	6.271E2 (1.034E2)
	T2	**2.125E3**+ (2.946E2)	9.116E3– (7.126E3SS)	3.916E3– (7.136E2)	3.643E3 (3.767E2)

“*+” and “−” denote the algorithm statistically significant better and worse than MFEA, respectively*.

As can be seen from Table 1, MTO-FWA shows obvious advantages in the average objective value of all the tasks in the classic MTO test problems compared with the basic MFEA. Compared with MFPSO and MFDE, MTO-FWA also shows better performance on both 15 out of 18 tasks, respectively, in the classical single-objective MTO test suite. The above statistical results verify the competitiveness and potential of the MTO-FWA algorithm in solving single-objective MTO. It is worth emphasizing that MTO-FWA reveals better performance than other EMT algorithms in most low and medium similarity test problems such as CIMS, CILS, PIMS, PILS, NIMS, and NILS. It is mainly due to the fact that the proposed TS can provide useful direction and step size and reduce the probability of negative information transfer by using information about the entire population rather than individual individuals. MFEA cannot mitigate the impact of negative knowledge transfer, which leads to the crossing process randomly happening with a lot of noise. Compared to MFPSO, MTO-FWA achieved better results on NIMS and NILS problems, because TV integrates information about the many sparks around the fireworks; therefore, it can provide better directions than the vector in PSO. Compared with MFDE, the MTO-FWA achieved better results on CIMS, CILS, PILS, and NIMS problems. It can be considered that the information used is the difference between two or more randomly selected individuals in DE, which is unpredictable. The information used in MTO-FWA comes from the difference between the two populations, so it is more specific.

### Experiments on MTO for Multiobjective Problems

Similar to the above study for single-objective MTO, this experimental study considers the nine multiobjective multitask problems built in the recent technical report (Yuan et al., 2017). Analogously, the test problems can be classified as high similarity (HS), medium similarity (MS), and low similarity (LS), three categories according to the similarity in the fitness landscape, and each category can be divided into three sub-categories, complete intersection (CI), partial intersection (PI), and no intersection (NI) by the degree of intersection of the value of optima in each dimension. Each MTO problem consists of two MOO problems, each consisting of two or three objective functions commonly studied in the literature. Meanwhile, the proposed MOMTO-FWA is also compared with the well-known NSGA-II (Deb et al., 2002), since it is frequently applied as the underlying basic solver by many multiobjective EMT algorithms. For a fair comparison, the population number for a single task is set to 100, and the maximum number of fitness evaluation for a single task is set to 100,000, using the average results of 30 independent runs for comparison. Both MOMFEA and NSGA-II use SBX, and polynomial variations use the same parameter values. In SBX, *p*_*c*_ and η_*c*_ are set to 0.9 and 20, respectively. As for polynomial mutation, *p*_*m*_ and η_*m*_ are set to *1/D*^6^ and 20, respectively.

Table 2 shows the average and standard deviation of the IGD of all algorithms that run 30 times independently on the classical multiobjective MTO test suite. The superior average IGD values are highlighted in bold. The Wilcoxon rank sum test is performed at the significance level of 5%, and the proposed MOMTO-FWA is compared with other multiobjective EMT algorithms. Significantly better and worse results than the basic MOMFEA are presented as “+” and “−.”

**Table 2:** Averaged value and standard deviation of the IGD obtained by MOMTO-FWA, MOMFEA, and NSGA-II on the multiobjective multitask problem.

		**MOMTO-FWA**	**MOMFEA**	**NSGA-II**
CIHS	T1	**2.437E−4**+ (5.507E−5)	3.422E−4 (9.643E−5)	1.733E−3– (2.345E−4)
	T2	**2.757E−4**+ **(8.144E−5)**	2.339E−3 (5.491E−4)	4.418E−3– (6.989E−4)
CIMS	T1	1.066E−1– (1.303E−2)	**5.932E−2** (7.136E−2)	1.306E−1– (5.421E−2)
	T2	1.263E−2– (9.682E−3)	**1.259E−2** (9.080E−3)	2.714E−2– (1.589E−2)
CILS	T1	**1.466E−4**+ (1.013E−5)	2.701E−4 (2.943E−5)	2.524E−1– (6.195E−2)
	T2	**1.448E−4**+ (6.575E−6)	1.867E−4 (8.093E−6)	2.022E−4– (8.687E−6)
PIHS	T1	**3.186E−4**+ (9.145E−5)	8.317E−4 (1.179E−3)	1.0581E−3– (3.854E−4)
	T2	**3.424E−4**+ (1.470E−4)	4.091E−2 (1.885E−2)	5.480E−2– (2.087E−2)
PIMS	T1	**7.767E−4**+ (3.510E−4)	2.862E−3 (1.257E−3)	5.033E−3– (1.367E−3)
	T2	**1.094E1**+ (3.423E0)	1.388E1 (4.159E0)	1.559E1– (3.700E0)
PILS	T1	4.307E−4– (6.266E−4)	3.495E−4 (3.003E−4)	**2.209E−4**+ (1.357E−4)
	T2	**3.834E−4**+ (1.044E−4)	1.109E−2 (2.350E−3)	6.343E−1– (5.097E−4)
NIHS	T1	**1.465E0**+ (1.072E−2)	1.552E0 (1.469E−2)	9.376E1– (7.172E0)
	T2	**2.709E−4**+ (6.558E−5)	4.961E−4 (1.058E−4)	8.450E−4– (1.731E−4)
NIMS	T1	**1.571E−1**+ (6.445E−2)	2.133E−1 (2.352E−1)	5.846E−1– (5.182E−1)
	T2	**2.623E−3**+ (1.667E−3)	3.541E−2 (6.654E−2)	6.518E−2– (5.992E−2)
NILS	T1	1.574E−3– (1.121E−3)	8.351E−4 (5.645E−5)	**8.277E−4**+ (5.807E−5)
	T2	**3.827E−3**+ (5.133E−4)	6.432E−1 (4.165E−4)	6.422E−1+ (3.896E−4)

“*+” and “−” denote the algorithm statistically significant better and worse than MOMFEA, respectively*.

As can be seen from Table 2, MOMTO-FWA shows obvious advantages in the average IGD value on 14 out of 18 tasks in the classic multiobjective MTO test problems compared with the basic MOMFEA. Compared with NSGA-II, MOMTO-FWA also shows better performance on 16 out of 18 tasks in the multiobjective MTO test suite. It is worth emphasizing that MOMTO-FWA reveals better performance than other multiobjective EMT algorithms in most low and medium similarity test problems such as CILS, PIMS, PILS-T2, NIMS, and NILS-T2 problems. Compared to MOMFEA, MOMTO-FWA achieved better results on CIHS, CILS, PIHS, PIMS, PILS-T2, NIHS, NIMS, and NILS-T2 problems, Even if it cannot surpass the performance of MOMFEA on CIMS, PILS-T1, and NILS-T1 problems, the performance of MOMTO-FWA is not much different. This may be because MOMFEA uses non-dominant ranking, while MOMTO-FWA uses S-metric as the evaluation index. In the later stage of the algorithm, the archiving-based mechanism reduces the diversity of solutions. Encouragingly, MOMTO-FWA achieves much better results than MOMFEA and NSGA-II on PIHS-T2, PIMS-T1, PILS-T2, NIMS-T2, and NILS-T2. It can be considered that the knowledge learning from simple tasks provides inspiration for solving difficult tasks and thus improves accuracy.

**Figure 3:**
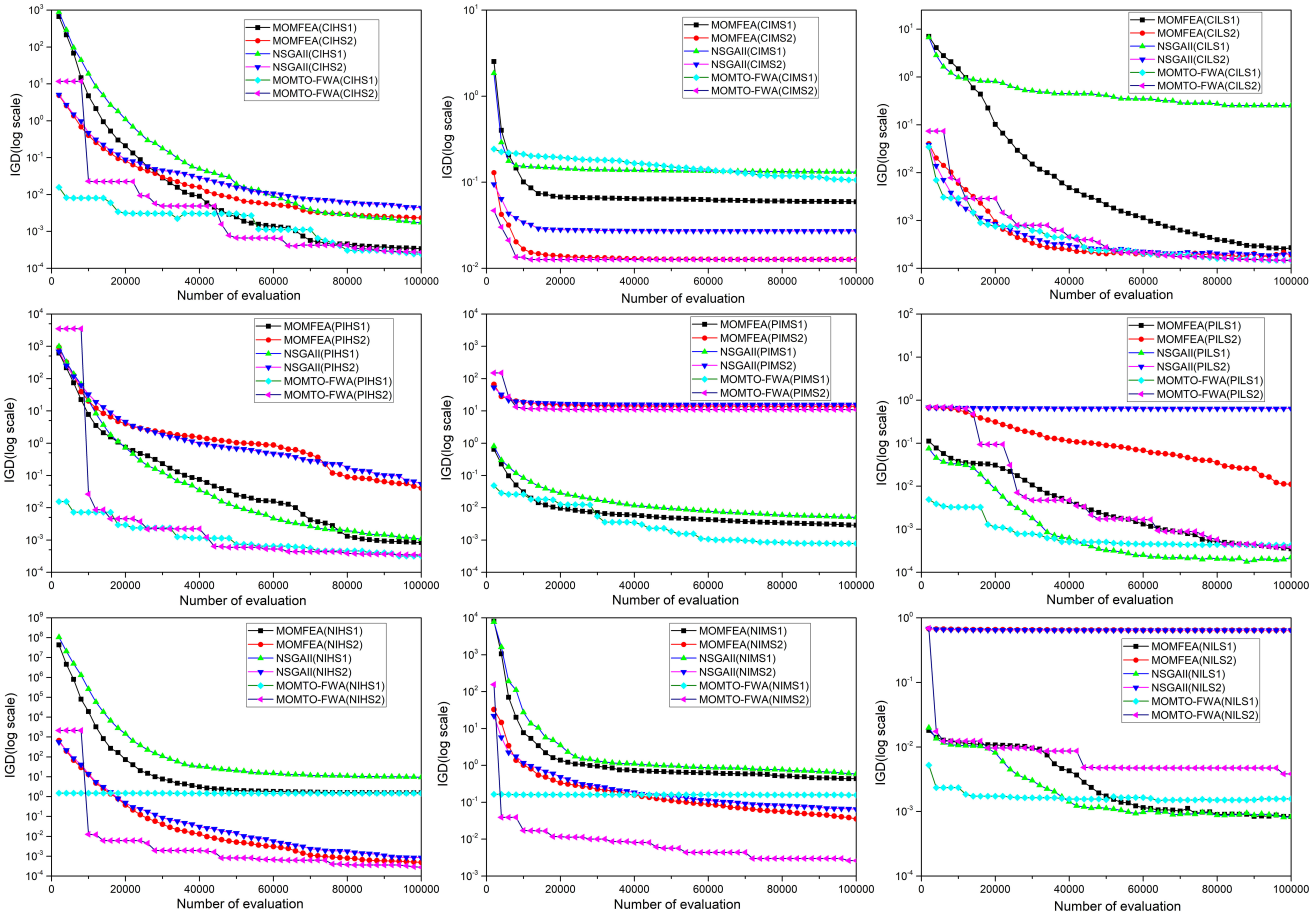
The average IGD with the number of evaluation for MOMFEA, NSGA-II, and MOMTO-FWA on the multiobjective multitask benchmark problem.

Figure 3 shows the average IGD values of MOMFEA, NSGA-II, and the proposed MOMTO-FWA after 30 independent runs on the classic multiobjective multitask test set. It should be noted that to indicate the changes in IGD more clearly, the starting point of the evaluation in Figure 3 starts from the 2000th evaluation, not from the 0th evaluation. Therefore, the algorithm has a preeminent starting point on some test problems, which does not mean that the random initialization of the population has undergone artificial intervention, but the population has converged to a state with a better IGD value within 2,000 evaluations. It is obvious from Figure 3 that the proposed MOMTO-FWA has terrific exploration ability and can quickly find out a better solution when the value of the fitness function of the initial population is terrible. In all the test problems, MOMTO-FWA is always on top in terms of IGD value within 20,000 evaluations. Besides, the proposed MOMTO-FWA converges faster than MOMFEA and NSGA-II in most problems.

## Conclusion

In this paper, we propose the strategy named TS to enable the FWA to solve MTO problems. The core idea is to bind a firework and its generated explosion sparks and GS into a task module to solve a specific problem. Through the performance of other task modules, a TS is generated around the firework to transfer the implicit genetic information between tasks. For the single-objective MTO problem, the objective function value corresponding to the task is used as the indicator to measure the performance of the task module to control the number of explosion sparks and the explosion amplitude. For multiobjective multitask problems, S-metric is applied to evaluate individual performance. The evaluation method based on the indicator is simple and effective, which is unified for utilizing the FWA to solve the SOO and MOO in MTO. Experimental results have shown that the proposed MTO-FWA can get promising results compared with the state-of-the-art multitask evolutionary algorithms on both SOO and MOO. There are several future research directions. One direction is to improve the efficiency of information sharing and transfer between fireworks. In addition, our current research focuses on the numerical optimization of two tasks. The many task problems and the simultaneous optimization of discrete and numerical tasks are the focus of the next phase of our research.

## Data Availability Statement

The datasets generated for this study are available on request to the corresponding author.

## Author Contributions

ZX: code implementation and writing the experiment. KZ, JH, and XX: guidance, revision of paper, and discussion.

### Conflict of Interest

The authors declare that the research was conducted in the absence of any commercial or financial relationships that could be construed as a potential conflict of interest.
